# The Leper Hospitals of the West Indies

**Published:** 1898-07-23

**Authors:** Louis Vintras

**Affiliations:** Physician to the French Hospital


### The Institutional Workshop.

### THE LEPER HOSPITALS OF THE WEST INDIES.

By Louis Vintras, M.D. Durh., B.Sc. Paris, Physician to the French Hospital.

III.

Of the leper settlement in Antigua, the principal island of this group, my visit to which was greatly facilitated by the courtesy of my friend Dr. Peraiz, there is hardly a word of good to be said, for here we find many of the more objectionable features which used formerly to be associated in the popular mind with all leper establishments. That such a state of affairs should be allowed to exist in the principal of the Leeward Islands, that is under the very eyes of the Government, is anything but creditable to those responsible for the disposal of the public money. For what little has been done much credit is due to Dr. Peraiz, and every sympathy will be felt for him in his struggle on behalf of humanity with the supineness of the Government authorities. Many would have preferred to dissociate themselves from such a state of affairs, but Dr. Peraiz has understood that such a course would be a betrayal of the trust confided to him, and prefers to stand by the institution until, by dint of perseverance, he shall have retrieved it from its present miserable condition.

In Antigua the fear of the leper is very vivid, but until lately the sufferers were allowed about the town, where they could dispose of small articles of basket work and other items, which they had made themselves. The inhabitants, however, seemed to have objected, and now these poor outcasts are sequestered on part of a hill, known as Rat Island, and situated at the entrance of the large estuary which forms the harbour of St. John's. On the top of the same hill the asylum for the insane is situated. Rat Island is in reality a small peninsula attached to the main island by a narrow strip of land, half way across which is a gateway which marks the limit of the leper's domain.

Here, then, in three sheds, for they are little better, about sixty lepers belonging to both sexes, live more or less promiscuously; they are separated at night, but live in common during the day. The interior of the wards (?) is in a very dilapidated condition, and devoid of any comfort; that the wards are kept clean is all that can be said, and the same of the patients, whose general appearance is miserable, but who, strange to say, keep up their spirits wonderfully under these depressing surroundings. This is due almost entirely to the kindly care of the matron of the asylum, a young lady, who for the last four years has devoted what time she can spare from her duties among the insane to comfort and cheer these poor outcasts, and who has only too evidently gained their gratitude and goodwill.

The patients are never allowed out when once they have entered the settlement. Each man is given a little patch of land which he cultivates, he being paid a small sum for what he produces. The women are also provided with some suitable work.

I was unavoidably prevented from visiting the Lepers' Home in Jamaica, and must, therefore, content myself with giving here the following facts gleaned from the last report of Dr. T. Foley Donovan, the medical attendant.

The home can accommodate 165 patients, and is under the control of an honorary superintending medical officer; there are also a superintendent who acts as dispenser, a matron, and a staff of trained nurses.

"The buildings," the report states, "are in an excellent state of repair, excepting some of the roofs, which require slight patching here and there and some minor repairs to wood-work. During the year, ending March, 1897, a new ward to accommodate eighteen females has been completed and handed over. The accommodation for females was taxed to its utmost capacity, and in view of the Leper Law of 1896 it was deemed advisable to provide more accommodation at the female side. An additional room was added to the matron's quarters; the paths and roads have been thoroughly macadamised; the surface-drains have been put in excellent order, and the enclosure of the male side of the institution with a concrete wall has been completed, which will contribute materially, if not effectively, to put an end to the surreptitious intercourse between the inmates and the outside public, and prevent the bartering of food which went on and which it was found impossible to check. The above new works and the necessary repairs were carried on in the usual prompt and efficient manner by the Public Works Department." Attention is called to the deficiency of the cubic space and the bad ventilation of the cells provided for the confinement of dangerous leper lunatics.

Dr. Donovan lays great stress on the question of occupation, and at this home much seems to be done in that direction, while games and entertainments are also got up; this is excellent, and we sincerely compliment those engaged in such useful work. If segregation is to be the order of the day, it is leper homes we require, and not leper gaols.

A hundred and thirty-six patients were treated during the year, while the daily average number in the home was 95·22. I cannot find in the report any financial statement.

The public leper asylums at Mahaica, Demerara, are organised more on the plan of settlements than of hospitals; large wards being only used for those lepers who are very bad or suffering from some other disease or complication, the others living in little detached cottages dotted all over the extensive grounds, some of which look quite picturesque, surrounded by their plots of ground full of all kinds of flowers and vegetables. This is by far the best way of dealing with leper patients, for in this large village settlement they can move at ease and lose the sense of sequestration. Again, being only a few together in each cottage, they take pride in keeping their cottages in better order and cleaner than that of their neighbours, and the results in this direction are quite striking. The polish of the floors, all done by hard rubbing, the walls well white-washed, the clean coverlets on the beds, and trim, I was going to say, military appearance of these interiors, is really a great achievement on the part of those at the head of these asylums, for the leper is not by nature disposed to cleanliness, either in his person or in his surroundings. Mahaica was the first leper settlement I visited, and it came to me quite as a revelation, for there where I had expected the unsavoury surroundings generally associated with this malady, I found everything bright, clean, and cheerful. The large wards are well ventilated and well kept. The shed under which the male inmates have their meals is hardly in keeping with the remainder of this establishment, and is both shabby and devoid of any air of comfort; but this, I understand, is only temporary.

The asylums are two in number, the male one at Mahaica, and the female asylum a little distance from it at Gorchum. At the end of March, 1897, there were 275 male inmates and 88 female. There had been 84 admissions during the year—70 males and 14 females—while there were 32 males and 4 females discharged, and the number of deaths was 44. The daily average number of inmates was 369.

The expenditure for the year ending March 31st, 1897, was 21,527 dollars, a reduction on the previous year, I understand, and by no means a high figure when we consider the excellent use to which the money is put.

Dr. Neal, the active medical superintendent, who has devoted his best energies to the improvement of this institution, attaches great importance to the occupation of leper patients, and in his last report gives the following interesting details: A farm is attached to the male asylum and is worked exclusively by the lepers, and has recently been enlarged, so successful has the venture proved. Of farm produce the following figures give the results of year's crops: 2,554 pounds of plantains, 3,641 pounds of sweet potatoes, 529 pounds of ochroes, and 119 pounds of egg plant. Financially speaking, however, the farm can hardly be called a success, for the produce thus grown only resulted in a saving of 64 dollars; but, even if it were a dead loss, the beneficial effect of the work on the inmates would in itself be sufficient reason for continuing the experiment. The report continues: "From the tailor's shop 1,348 articles of clothing were made, and repairs in excess of this, throughout the year. The shoemaker with his assistants, all inmates, turned out 291 pairs of slippers, 65 pairs of shoes, and 38 pairs of boots. The females at Gorchum make all their own clothing, there being no paid seamstress, and the inmates of both asylums mostly do their own washing. . ."

Elsewhere we find in the report for the same year, and addressed to the Surgeon-General of the Colony, that "the general behaviour has been good. Absconding and going out when they want to is not unfrequent; if, however, there were no enticements by their friends or relatives, and the outside public did not assist them, it would cease; moral persuasion before and punishment after is not a strong enough deterrent. . . . School is held regularly at both asylums, inmates being the instructors."

The male asylum is situated by the sea, just at the mouth of a river, and, though part of the grounds is below the sea level, the position is as good as can be obtained in a country so peculiarly situated. Sea-bathing is indulged in and encouraged, and has a marked influence on the general condition of the inmates. The hospital at Mahaica is in every way worthy of the great colony of British Guiana, and, though still amenable to improvement, it might well serve as a model for the establishment of similar institutions, and the progress which has been made of late years reflects the utmost credit on Dr. Neal.

It will thus be seen that there is a great difference in the way in which lepers are treated in these sister colonies, and while in some places they are absolute prisoners, in others they are allowed to leave if they desire to do so; here they are treated as dangerous animals, and literally caged up; there they are kindly thought of, and made to understand that it is their own benefit to be in an institution where they can be tended and cared for and the disease checked in its progress.

The great difference in the way lepers are treated arises from the fact that the controversy about the contagiousness of leprosy is still raging, and that no definite opinion has yet been arrived at. It would be obviously out of place here to enter into any discussion of the subject, but I may be allowed the following remarks concerning the much-vaunted advantages of absolute segregation, which many medical men are so urgently pressing home to the authorities in the West Indies.

It must be remembered that in applying a measure so severe as the absolute privation of liberty to a comparatively large class of individuals scattered over different parts of the globe, we have other things to consider than the mere cowardly instinct of protecting ourselves against them. What have these people done that we should imprison them for life? Yet that is what segregation carried to its extreme means, and what a section of the medical profession are now asking for. Have we a right to inflict punishment on any individual because he is stricken with a terrible disease, thus adding punishment to punishment? It is all very well for science to be invoked, but science has no moral responsibility; while those who have become her high priests and priests have, and though any particular theory may be scientifically correct, the putting of it into practice may prove a very immoral action. "The end justifies the means," it is said, perhaps, but the end does not justify those who employ the means. The welfare of the majority is an excellent argument, but, after all, merely the argument of the strongest. I do not, however, wish to speak against segregation in the case of leprosy, but merely to express a doubt whether we are justified in carrying it to extremes, and to point out one aspect of a question which is much broader than the contagion of leprosy. If, however, we resort to such a step, it is certain that we owe these poor fellow-creatures every compensation in the way of comfort and kindness, which will at least excuse our action, if it cannot justify it; for we cannot even cherish the thought so far, that it is for their welfare, beyond the fact that they are attended to, their sores dressed, and good food supplied them; for so hopeless is the idea of a cure for the present that no special treatment is carried out in any of these institutions, and the lepers are never treated for leprosy, but merely for intercurrent diseases and complications. Segregation is for the well-being of the healthy, whatever sophistry may be written about it, and we cannot say at the present day that we have in any way done our duty towards these most miserable of human beings.

